# Biocompatible Gels of Chitosan–Buriti Oil for Potential Wound Healing Applications

**DOI:** 10.3390/ma13081977

**Published:** 2020-04-23

**Authors:** Maria Onaira Gonçalves Ferreira, Idglan Sá Lima, Alessandra Braga Ribeiro, Anderson O. Lobo, Marcia S. Rizzo, Josy Anteveli Osajima, Leticia Miranda Estevinho, Edson C. Silva-Filho

**Affiliations:** 1 Materials Science and Engineering Graduate Program, Federal University of Piauí, Campus Universitário Ministro Petrônio Portella, Teresina, PI CEP 64049-550, Brazil; mariaonaira@hotmail.com (M.O.G.F.); i.dglan@hotmail.com (I.S.L.); lobo@ufpi.edu.br (A.O.L.); marciarizzo@ufpi.edu.br (M.S.R.); josyosajima@ufpi.edu.br (J.A.O.); 2 Faculty of Biotechnology, CBQF–Centre of Biotechnology and Fine Chemistry–Associate Laboratory, Catholic University of Portugal, Rua Diogo Botelho 1327, 4169-005 Porto, Portugal; abribeiro@porto.ucp.pt; 3 Polytechnic Institute of Bragança, Agricultural College of Bragança, Campus Santa Apolónia, 5301-855 Bragança, Portugal; leticia@ipb.pt

**Keywords:** polysaccharides, biological, healing

## Abstract

The buriti oil (*Mauritia flexuosa* L.) can be associated with polymeric matrices for biomedical applications. This study aimed to evaluate the effect of chitosan gel (CG) associated with buriti oil (CGB) as a healing agent. The fatty acids and volatile compounds composition of buriti oil were performed and the composite gels were characterized using FTIR and thermal analysis. Biological tests including antimicrobial, antioxidant, anti-inflammatory and healing effects were also investigated. Buriti oil is composed of oleic and palmitic acids, and the main volatile compounds were identified. The buriti oil did not show antimicrobial activity, on the other hand, the composite gel (chitosan and oil) proved to be efficient against *Staphylococcus aureus* and *Klebsiella pneumonia* at the 10 mg/mL. Similar behavior was observed for antioxidant activity, determined by the β-carotene bleaching assay, composite gels presenting higher activity and buriti oil showed anti-inflammatory activity, which may be related to the inhibition of the release of free radicals. Regarding wound healing performed using in vivo testing, the composite gel (CGB) was found to promote faster and complete wound retraction. The results indicated that the gel chitosan–buriti oil has a set of properties that improve its antibacterial, antioxidant and healing action, suggesting that this material can be used to treat skin lesions.

## 1. Introduction

Chitosan is a biopolymer that has been widely studied to be used in several areas, as it has biocompatibility, biodegradability, non-toxicity, mucoadhesion, and antimicrobial activity [1]. It has been used in tissue engineering [2], dressings, accelerating healing [3,4], wound care [5], and other biomedical and pharmaceutical applications [6].

Chitosan gel has to show potential to be used as a wound healing system. Ideal properties include: the ability to stimulate the proliferation of fibroblasts,; to inhibit the growth of inflammatory cells; to act against bacteria and fungi; to stimulate the activation of macrophages and neutrophil migration; to promote re-epithelialization; to provide a lower degree of fibroplasia; to stimulate the immune system; to be oxygen permeable; low-toxicity; promote bioadhesion, to have hemostatic potential; and to have antitumor action. All these properties contribute to medical and pharmaceutical application [7,8,9,10,11,12].

The wound healing process involves the stages of hemostasis, inflammation, migration, proliferation, and cell maturation [13]. The presence of infections and contamination by bacteria can delay this process, therefore it is necessary to use medicines that keep the place moist and that allow gas exchange, and also the use of dressings that are easily removable or absorbable [13,14].

Chitosan gel associated with other substances may acquire new properties that optimize and extend its applicability and efficiency in wound healing [3]. In this work, the substance associated with the chitosan gel was buriti oil (*Mauritia flexuosa L*.), which presents an adequate profile in bioactive and antioxidant compounds, and it can be further explored properly by regional industries for the development of products.

Antioxidants comprise a set of different substances such as vitamins, minerals, natural pigments, and other plant compounds as well as enzymes that block the effect of free radicals. Oxidative stress is mainly responsible for damage and molecular changes [15].

The harmful effects of oxidative stress include the oxidation of biomolecules such as cell membrane lipids, tissue proteins or enzymes, carbohydrates, and DNA, leading to damage that from a certain level becomes irreversible [15]. The provision of exogenous antioxidants may be essential for maintaining antioxidant protection. Therefore, it is believed that skin wounds heal faster if the antioxidant properties of buriti oil are combined with the beneficial properties of chitosan gel.

The low cost and biological potential of these natural products, chitosan gel and buriti oil contribute to the development of new products that accelerate the healing process, preventing undesirable effects. In this context, this study aimed to evaluate the antimicrobial, antioxidant, anti-inflammatory, and healing activity of chitosan gel associated with buriti oil.

## 2. Materials and Methods

### 2.1. Materials

The chitosan of medium degree of deacetylation (95%) obtained from Primex, buriti oil was purchased at local markets in the city of Bom Jesus, State of Piauí, Brazil. The local population extracts oil from the fruit of the Buriti by heating the fruit in water in an artisanal manner without the use of solvents. The reagents used were: sodium carbonate (MERCK, Darmstadt, Germany), folin (MERCK, Darmstadt, Germany), methanol (CARLO ERBA, Val-de-De-Reuil, France), ethanol, β-carotene (SIGMA-ALDRICH, St. Louis, MI, USA), linoleic acid (FLUKA, Milan, Italy), tween 40 (ACROS ORGANICS, Morris Plains, NJ, USA), chloroform (ABSOLVE, Odivelas, Lisbon, Portugal), dichloromethane (MERCK, Darmstadt, Germany), aluminum chloride (MERCK^,^ Hohenbrunn, Germany), acetic acid (VETEC, Jaraguá do Sul, Santa Catarina, Brazil), sodium hydroxide (DINÂMICA, Indaiatuba, São Paulo, Brazil), butylated hydroxyanisole-BHA (ACROS ORGANICS, Morris Plains, NJ, USA ), quercetin (SIGMA-ALDRICH, St. Louis, MI, USA), Brain Heart Infusion (HIMEDIA, Munbai, India), Mueller Hinton agar (HIMEDIA, Munbai, India), sodium chloride (IMPEX, Wood Dale, IL, USA), sodium sulfate (FLUKA, Steinheim, North Rhine-Westphalia, Germany), hyaluronidase enzyme (350 units) ((SIGMA-ALDRICH, St. Louis, MI, USA), and potassium salt of human umbilical cord hyaluronic acid (SIGMA-ALDRICH, St. Louis, MI, USA). All the regents were 98% and 99.9% pure. The water was MilliQ^®^ (Millipore Corporate, Burlington, MA, USA) deionized. All the reagents were used without prior purification.

### 2.2. Gels Synthesis

Pure chitosan gel (CG) was obtained from the reaction of chitosan power with 2.0% aqueous acetic acid solution, 3.0 g of chitosan were used for 100.0 mL of acetic acid solution. The solution was stirred for 30 min. To obtain the chitosan gel with buriti oil (CGB), 1.0 mL of the oil was added to the gel and stirred for 10 min.

### 2.3. Characterizations

#### 2.3.1. Thermal Analysis (TG) and Infrared Spectroscopy (FTIR)

The thermal stability study of materials was performed by the TG (thermogravimetry) technique. The thermogravimetric curves were obtained by the SDT Q600 V20.9 Build 20, model DSC-TGA Standard (New Castle, DE, USA), with a heating ratio of 10 °C/min, under nitrogen atmosphere, alumina sample holder, in the temperature range from 273 to 600 K, and 10 mg of sample mass approximately. Infrared spectra of materials and composite gels were obtained using the MB Series Bomem FTIR spectrometer in 32 scans in the region of 4000–600 cm^−1^ with a resolution of 4 cm^−1^ and 32 accumulations by ATR (Brucker Optics-Vertex 70, Brucker, Billerica, MA, USA).

#### 2.3.2. Scanning Electron Microscopy (SEM)

The micrographs of lyophilized gels were performed on the field emission scanning electron microscope (SEM) with field emission gun, FEI brand, Quanta FEG 250 model, with acceleration voltage from 1 to 30 kV, equipped with Ametek SDD (Silicon drift detectors) EDS, model HX-1001, Apollo X-SDD detector (FEI Company, Eindhoven, The Netherlands). Samples were fixed on double-sided carbon tape, grounded with silver adhesive paint and covered with Quorum model Q150R Aunametalizer (Laughton, East Sussex, UK) for 30 s at 20 mA by plasma generated in argon atmosphere.

#### 2.3.3. Minority Volatile Compounds (MS)

Minority volatile compounds were analyzed by GC-MS. The internal standard was 3.0 µL of buriti oil added in 10.0 mL dichloromethane and 100.0 µL of 4-nonanol (24.012 µg/mL), the mixture was kept refrigerated for further analysis. A Varian 3800 gas chromatograph (VARIAN, Palo Alto, CA, USA) with a 1079 injector and a Varian Saturn 2000 ion-lock mass spectrometer (VARIAN, Palo Alto, CA, USA) was used. Injections of 1.0 µL were performed in splitless mode (30 s) on a Sapiens-Wax MS column (30 m; 0.15 mm; 0.15 µm film thickness, Teknokroma, Barcelona, Spain). The carrier gas was helium 49 (PRAXAIR, Danbury, CT, USA)) at a constant flow rate of 1.3 mL/min. The detector has been set to electronic impact mode with ionization energy of 70 eV, a mass acquisition range of 35 to 260 m/z and an acquisition range of 610 ms. The oven temperature was initially set at 60 °C for 2 min and then increased from 60 to 234 °C at a rate of 3 °C/min, increased from 234 to 260 °C at 5 °C/min and finally kept at 260 °C for 10 min. Injector temperature was 250 °C with 30 mL/min split flow. The compounds were identified using MS Workstation software version 6.9 (Varian), comparing mass spectra and retention indices with those of pure standards. The minor compounds were quantified as 4-nonanol.

#### 2.3.4. Fatty Acids

For fatty acids analysis buriti oil, it was used, esterification process to obtain the fatty acid methyl esters. The fatty acid separation was performed on a GC1000 DANI gas chromatograph (DANI, Cologno Monzese, Italy) equipped with a Marcherey-Nagel Optima^®^ column flame ionization detector (FID) (MACHEREY-NAGEL, Düren, North Rhine-Westphalia, Germany, 25 m long and 0.32 mm in diameter, 0.25 µm film. The carrier gas was hydrogen, flow 4 mL/min. Initial heating was 50 °C for 2 min, 30 °C/min to 125 °C, 5 °C/min to 160 °C, 20 °C/min to 180 °C, 3 °C/min to 200 °C, 20 ° C/min to 220 °C, and finally this last temperature was kept for 15 min.

Fatty acids were identified by comparison of retention times with standards (Supelco^TM^ 37 Component FAME Mix, Merck KGaA, Darmstadt, Germany) and quantified by area normalization, followed by obtaining the percentage of each fatty acid.

### 2.4. Antibacterial Tests

#### 2.4.1. Bacterial Lineage

Standard strains of Gram-positive and Gram-negative bacteria namely *Staphylococcus aureus* (ATCC 43300) and *Klebsiella pneumoniae* ATCC 13883 were used, respectively, and both strains were supplied by the Microbiology Laboratory of the Bragança Polytechnic Institute (IPB)—Portugal.

#### 2.4.2. Preparation of the Microbial Suspension

Cultures were obtained by transferring an initial of bacterial growth on nutrient agar to an Erlenmeyer 15.0 mL of Brain Heart Infusion (BHI) medium, followed by incubation at 37 °C under shaking overnight. The bacterial inoculum used in the bioassays was prepared by transferring aliquots of the culture to a falcon tube containing 9.0 mL saline (0.9% *v*/*v*). The absorbance of inoculum suspension was measured at 540 nm and achieved 0.3–0.4.

#### 2.4.3. Determination of Minimum Inhibitory Concentration (MIC)

The minimum inhibitory concentration (MIC) was determined using a microdilution method a suspension of 1.5 × 10^8^ colony-forming units per mL (CFU/mL), according to the method for diluting antimicrobial susceptibility testing for aerobically growing bacteria [16]. It was tested pure gel and composite gel at concentration from 0.156 to 40 mg/mL and buriti oil at concentration from 2.5 mg/mL to 320 mg/mL. In addition it was performed negative and positive controls, all microplates were incubated at 37 °C for 24 h. MIC was defined as the lowest concentration of the sample capable of inhibiting microbial growth, as indicated by the TTC staining.

### 2.5. Antioxidant Activity

#### Β-Carotene Bleaching Test

The β-carotene bleaching assay was performed as described by Koleva et al. (2002). To each of 0.0010 g of β-carotene was added 5 mL of chloroform, from this solution a 1.5 mL aliquot was taken and mixed with 0.033 g of linoleic acid and 0.3 g of Tween 40. The chloroform was evaporated using a rotary evaporator (R-210, Buchi, Flawil, Switzerland) at 50 °C for 15 min. The stock solution was resuspended in 75 mL of previously oxygenated MilliQ^®^ water, which was called the emulsion. The blank solution was prepared using 0.0022 g of linoleic acid, 0.0200 g of Tween 40.0 and 5.0 mL of previously oxygenated MilliQ^®^ water.

The antioxidant capacity was measured using 30.0 μL of the sample in 250.0 μL of emulsion. The control was prepared using 250.0 μL of emulsion in 30.0 μL of solvent (water or ethanol), and 250 μL of the blank solution was added to 30.0 μL of solvent. Absorbance (492 nm) was taken on a Thermo Fisher Scientific microplate reader (Waltham, MA, USA) in initial time and after 2 h. The samples are kept under stirring in the dark at 45 °C. The antioxidant activity (AA) was calculated using the following equation:

%AA = (1 − ((Absa_t0_ − Absa_t2_)/(Absctrl_t0_ − Absctrl_t2_))) × 100

where:

%AA = percentage of antioxidant activity

Absa_t0_ = Absorbance at the initial time

Absa_t2_ = Absorbance at the final time

Absa_t0_ = Control absorbance at the initial time

Absa_t2_ = Control absorbance at the final time

The antioxidant activity data of the material was expressed as µmol/L BHA equivalent.

### 2.6. Anti-Inflammatory Activity

The anti-inflammatory activity was evaluated spectrophotometrically, measuring the inhibitory effect of pure chitosan gel, composite gel and buriti oil on reactions catalyzed by hyaluronidase using the method described by Silva et. al, 2012 [17]. The materials were prepared with the hyaluronidase enzyme (Type IV-S) in a proportion of 50.0 μL of CG, CGB and buriti oil and 50.0 μL of the enzyme, with incubation at 37 °C for 20 min. Enzyme activation was done by adding calcium chloride (1.2 mL of 2.5 × 10^−3^ mol/L) in incubation at 37 °C for 20 min. After 0.5 mL of the sodium salt of hyaluronic acid (0.1 mol/L) was added and the mixture was incubated for 40 min at 37 °C. After that time, 0.1 mL of potassium tetraborate was added at concentration of 0.8 mol/L. The system was boiled in a water bath for 3 min. The mixture was cooled to 10 °C with the addition of 3.0 mL of p-dimethylaminabenzaldehyde and incubated at 37 °C for 20 min. Finally, the absorbance was measured at 585 nm. Water was used as a control and all tests were performed in triplicate.

### 2.7. Healing Assays

#### 2.7.1. Ethical Aspects

The research was conducted according to the recommendations described in “Guide for Care and Use of Laboratory Animals” (Institute of Laboratory Animals Resources, National Academy of Science, Washington, D.C., 2011). All animal procedures were guided by ethical principles established by the National Council for Animal Experimentation Control and the UFPI Ethics Committee for Animal Experimentation (207/16, 16th September 2016), according to the provisions of Law 11.794, 08th October 2008.

#### 2.7.2. Animals

Mice, Swiss strain (*Mus musculus*), albinos, adults, males, and females, from 2 to 4 months old, with body mass around 25–30 g, provenience from the Central Bioterium for Agrarian Sciences in the Federal University of Piauí/Brazil. The animals were kept under monitored conditions at a temperature equivalent to 26 ± 1 °C, with feed and water ad libitum, and a 12 h light/dark cycle. The animals were randomly divided into distinct groups of twelve specimens, according to a specific treatment, among the groups were, necessarily, the topical treatment with saline and collagenase ointment (negative and positive control groups, respectively), the pure chitosan gel (CG) and associated gel with buriti oil (CGB).

#### 2.7.3. Procedure of Wounds Excision

After intramuscular anesthesia in mice, composed of xylazine hydrochloride 0.04 mL/100 g and 10% ketamine hydrochloride 0.08 mL/100 g, dorsal region trichotomy was performed by circular removal of the tissue with skin and approximately 0.6 cm in diameter with the aid of a punch to form a skin wound with exposure of the dorsal muscle fascia. Finally, the animals were treated daily with an application of approximately 10 mg ointment, saline solution and 10 mg gels, saline solution and gels, being placed in their respective cages under observation. The non-sutured wounds were not covered with any kind of dressing.

#### 2.7.4. Wound Treatment and Evaluation

The wound treatment of each group was administered topically in the injured region, with the products assigned to each group. The wounds were cleaned with saline (0.9%) before the everyday new application of the test products (Ferreira et al., 2019). Euthanasia of three animals from each group was performed on 3rd, 7th, 14th, and 21st days and it was provoked by using pentobarbital overdose 10–15 mg/100 g of animal weight, intraperitoneally, followed by skin flap excision for histological analysis, and finish on 21st day of treatment.

#### 2.7.5. Macroscopic Evaluation of Skin Lesion in Mice

The animals treated with the active principles and the control groups under analysis were followed daily by observing the lesion repair, referring to the changes regarding the presence or absence of edema, exudate, and crust, and wound coloration. Digital photographic recording of the wound of all animals of the experimental groups was daily performed during the treatment, and the lesions measured with the aid of an analog caliper on 1st, 3rd, 7th, 14th, and 21st days of treatment.

#### 2.7.6. Qualitative Histopathological Evaluation

After euthanization, a skin fragment of the backs of three animals in each group was desiccated on 3rd, 7th, 14th, and 21st days of treatment, the size of this fragment was sufficient to cover the entire injured region (Ferreira et al., 2019). All skin lesion samples obtained were fixed in 10% buffered lkformaldehyde for 24 h and taken to a battery with a crescent sequence of alcohol for dehydration and diaphanized in xylol. Then, they were submitted in paraffin baths to 60 °C and embedded in wax blocks. Cuts of 5 µm thick were made with Leica (Buffalo Grove, IL, USA) microtome, stained with Hematoxylin-Eosin (H.E.) and samples of cutaneous tissue mounted between slide and coverslip. The slides were evaluated by light microscopy and the images digitally photographed. The reading aimed to observe the inflammatory and healing process through the parameters of the presence of granulation tissue, vascular proliferation, inflammatory infiltrate intensity, presence of collagen, and reepithelization.

## 3. Results and Discussion

### 3.1. Characterizations

#### 3.1.1. Thermal Analysis

**Figure 1:**
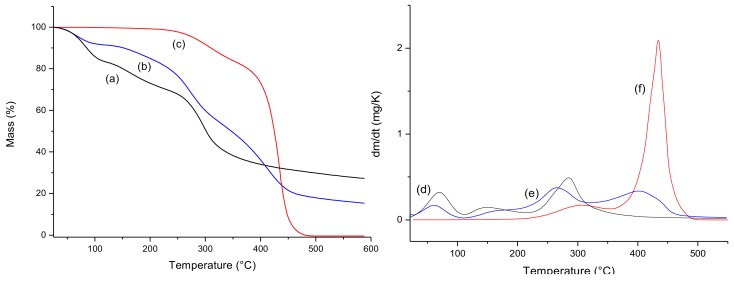
Thermogravimetric curves (TG) of pure chitosan gel (a), chitosan gel with buriti oil (b), and buriti oil (c), and Derivative Thermogravimetric curves (DTG) of chitosan gel (CG) (d), chitosan gel with buriti oil (e), and buriti oil (f).

Figure 1 shows the thermogravimetric curves of pure chitosan gels (CG) (Figure 1a), chitosan gels associated with buriti oil (CGB) (Figure 1b) and buriti oil (OB) (Figure 1c). For chitosan gel lyophilized (CG) (Figure 1a) three events were observed, best viewed in Figure 1d which shows the derivative of this curve. The first event referring to water and other volatile (until 100 °C), the second refers to the condensation of the hydroxyl and amine groups with the release of water and ammonia (100–200 °C) and the last event occurred from 220 °C with the thermal decomposition of the polysaccharide structure [18]. For the buriti oil (TG—Figure 1c and DTG—Figure 1f), two distinct events are observed. The first event referring to the condensation of superficial groups (250–350 °C) and the second refers to total thermal decomposition. The associated gel (CGB) (TG—Figure 1b and DTG—Figure 1e) was observed four events. The insertion of buriti oil in the gel caused a change in the thermal stability of the chitosan gel, which showed three events. It indicates that there was an interaction between the oil and Chitosan. The fourth event of associated gel CGB degradation at approximately 480 °C refers to buriti oil.

It can also be observed a shift in the initial and final temperatures of the chitosan gel containing buriti oil when comparing with the temperatures observed in the DTG’s of the starting materials, thus indicating that there is an interaction between chitosan and buriti oil.

#### 3.1.2. Infrared Spectroscopy

**Figure 2:**
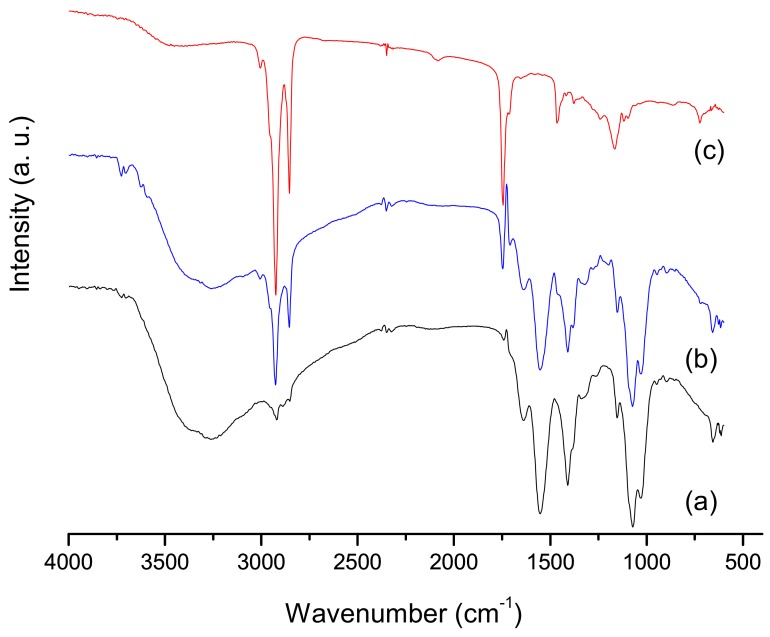
Infrared spectroscopy (FTIR) spectrum of chitosan gel (a), chitosan gel with buriti oil (b), and buriti oil (c).

Figure 2 showed the infrared spectroscopy (FTIR) graphics of pure chitosan gel, buriti oil, and associated gel (chitosan and buriti oil).

The infrared spectrum of pure chitosan gel lyophilized (CG), Figure 2a, has some characteristic bands such as the intense band from the N–H axial deformation and the OH stretch vibration of the hydroxyl structure in the region of 3550–3100 cm^−1^, which has enlargement caused by numerous interactions proving gel formation. Approximately at 1650 cm^−1^ appears the band from the C=O of amide axial deformation, at 1551 cm^−1^ appears the amine groups deformation and at 1465–1423 cm^−1^ there is the OH and CH_2_ deformation [4]. In the region between 1200–900 cm^−1^ there is the presence of a broad band referring to the polysaccharide structure of chitosan [19].

FTIR spectrum of buriti oil, Figure 2c, was observed a small band in a region above 3000 cm^−1^ referring to hydroxyl groups of acids present in its structure [19,20]. Besides, it was possible to observe the bands referring to the C–H bond of the methyl groups in the region of 2900 cm^−1^ for buriti oil. The 1700–1500 cm^−1^ regions were observed the presence of C=O of fatty acids of oil structure [19,20]. The graphics of CGB material, Figure 2b, was observed that the spectrum was a combination of the main bands characteristic of chitosan and buriti oil [3].

#### 3.1.3. Scanning Electron Microscopy

**Figure 3:**
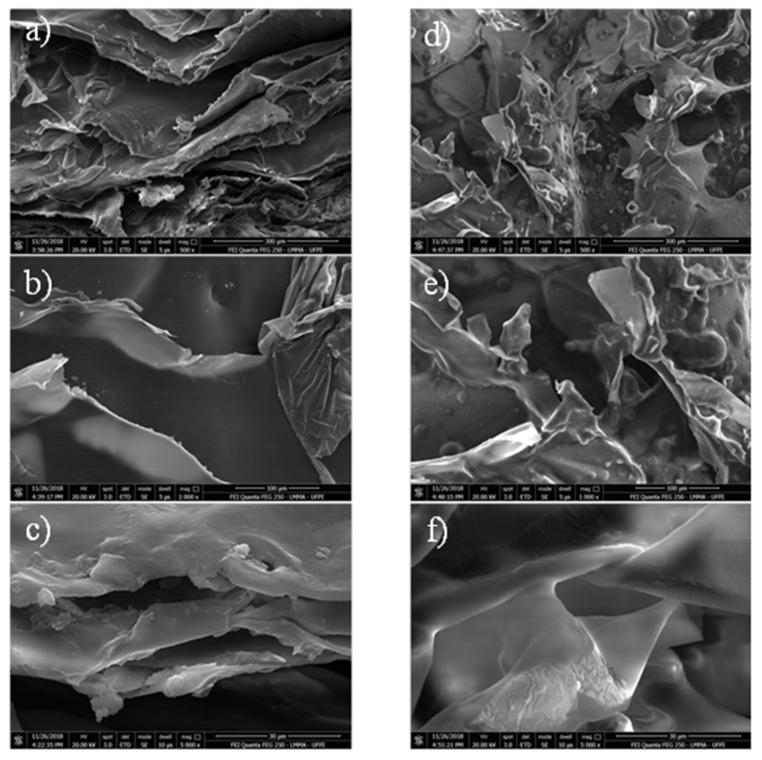
Scanning electron microscope (SEM) images of chitosan gel (CG) (**a**—300 μm, **b**—100 μm, and **c**—30 μm) and chitosan gel with buriti oil (CGB) (**d**—300 μm, **e**—100 μm, and **f**—30 μm).

Figure 3 showed the micrograph images of the synthesized materials after lyophilized, with three images (different magnifications) related to the chitosan gel (a–c) and the other three (d–f) referring to the chitosan gel with buriti oil. Both materials showed an irregular surface, however the CGB images showed oil deposits on the gel surface and higher porosity (Figure 3d–f). The porous nature of material may promote greater gas exchange and nutrient entry into cells, and it can contribute to vascularization, which is essential for healing [13]. In addition, rougher and porous materials help to adhere more easily to the skin and allow cell proliferation [21].

#### 3.1.4. Minority Volatile Compounds

The main volatile compounds identified in buriti oil are shown in Table 1. These compounds are responsible for the odors and aromas of the oils, many alcohols and esters are typical products resulting from oleic acid self-oxidation. Oleic acid is one of the components that participate in the construction of the cell membrane [22].

Phenethyl alcohol or 2-phenylethanol is a primary alcohol, also identified in buriti oil, has light sensitivity, and decomposes when exposed directly to air. The light sensitivity is offset by the presence of ionones, which are derived from the degradation carotenoids. In the case of buriti oil, β-ionone was identified, which is indicative of vitamin A activity, is soluble in fat and has antioxidant activity [23].

There is evidence that indicates that hexadecanoic and octadecanoic acids, presented in volatile compounds of buriti oil, promote antimicrobial and antioxidant activity [24,25]. Ethyl hexadecanoate is widely used by industry as a flavoring agent in food and pharmaceutical products [26].

#### 3.1.5. Fatty Acids

Table 2 showed the percentage of fatty acids composition of the buriti oil. The average degree of unsatured was 78.5%, the buriti oil has about 20% saturated fatty acids. In addition, to the main fatty acids, traces of capric acid, myristoleic acid, eicosapentaenoic acid, behenic acid, and ligonocenic acid were also verified.

Fatty acids are structural components of biological cells and act as precursors of intracellular messengers. These metabolites are capable of interfering with the inflammatory process and are related to lower free radical production [27].

Buriti oil is mostly composed of palmitic and oleic acid. Oleic acid is a yellow monounsaturated fat, also known as omega-9, the use of this unsaturated fatty acid may be related to strengthening the immune system and reducing inflammatory processes [26]. In addition, it is one of the constituents of the epidermis, being vital for cell membrane construction, and acting as a protective barrier to prevent skin dehydration. Oleic acid is responsible for the antioxidant activity of buriti oil as it protects cell membranes from free radical attack and is less susceptible to oxidative damage [23,28].

### 3.2. Antibacterial Activity

*Staphylococcus aureus* and *Klebsiella pneumoniae* were chosen since these bacteria are the most commonly species present in skin infections. These are opportunistic bacteria that can proliferate in the body whenever the immune system is depressed. Besides, its ability to resist against many drug therapies promotes an important challenge for the pharmaceutical industry, and for this reason, possible ways to overcome these resistance mechanisms have been investigated [29].

Buriti oil is a hydrophobic compound and has an affinity to the cytoplasmic membrane. When it diffuses into the wall and interacts with phospholipids, it can alter permeability and cause the loss of important bacterial materials and may influence microbial multiplication and survival. The oil concentrations tested (1.25–160 mg/mL) did not inhibit the growth of either *Staphylococcus aureus* ATCC (43300) or *Klebsiella pneumoniae* ATCC (13883). However, Park et al., 2009 found that the antimicrobial activity of this oil can be activated by sensitizing light to an appropriate wavelength.

Chitosan in the gel form presents protonated cationic groups, which can increase the electrostatic interaction of chitosan with the bacterial cell wall. The cell wall of bacteria, particularly of Gram-positive bacteria is essentially formed by teichoic acid bounded with peptidoglycans. This acid has phosphate groups promoting negative charge of the wall, the interaction between chitosan and phosphate groups destabilizes the bacterial wall and it may interfere with osmosis and biosynthesis. In the case of Gram negative, Alvarenga et al., 2007 [30], suggest that chitosan promotes disturbances in the metabolism of these microorganisms, causing death and inhibition of cell growth.

The synergism of the antimicrobial activity of chitosan gel and buriti oil was evident, which showed that the MIC was significantly lower for the composite gel than for the pure chitosan gel, and also the effect for this association was more pronounced for *K. pneumoniae* (Table 3). In fact, as mentioned before, buriti oil did not affect bacterial growth at the concentrations tested.

Our results suggest that a lower material concentration (CG with buriti oil) is required to inhibit the growth of *S. aureus* and *K. pneumoniae*, because the oil potentiated the antibacterial action of CG. The synergistic effect expands the possibilities of applications of this associated material. Costs could be reduced due to the high bioavailability of chitosan, but besides this, the antimicrobial activity is also important.

Bacterial resistance is the major concern of the pharmaceutical industry. The most of hospital infections are associated with the resistance of microorganisms to broad-spectrum antibiotics. Due to this problem, once again the potential of these gels is emphasized, since the combination of buriti oil can act quickly, not allowing the development of mechanisms of resistance even with intermittent applications [31,32,33].

### 3.3. Antioxidant Activity

The term antioxidant means that it prevents oxidation of other substances that occur in metabolic processes or exogenous factors such as ionizing radiation. In living organisms oxidative stress is mainly responsible for damage and molecular changes [15].

Reactive nitrogen species (RNS) are derived from nitric oxide (•NO). The term RNS not only includes free radicals such as •NO and nitrogen dioxide (•NO_2_), but also radical species known as peroxynitrite anion (ONOO^−^), peroxynitrous acid (ONOOH), and dinitrogen trioxide (N_2_O_3_). Both ROS and RNS, endogenously produced are essential to life and are involved in several biological functions, for example, energy production, phagocytosis, regulation of cell growth and signaling, synthesis of important biological compounds, and metabolism xenobiotics [15].

On the other hand, these reactive species can be harmful when their generation exceeds the ability of endogenous antioxidant defense mechanisms to remove them. The reactive species become harmful, causing oxidative stress through the oxidation of biomolecules such as cell membrane lipids, tissue proteins or enzymes, carbohydrates and DNA, leading to damage from a certain level become irreversible [15,34].

In the normal cellular environment, ROS and RNS are indispensable, however when production is excessive or when antioxidants are depleted these substances can become deleterious [15]. For this reason, it is necessary, for example, to provide exogenous antioxidants, which are essential for maintaining antioxidant protection. A high concentration of carotenoids, especially β-carotene, in buriti oil could offer this material a high oxidative stability, which could be used in the treatment of cutaneous wounds to neutralize naturally produced free radicals due to skin injury [26,35,36].

The antioxidant activity of pure chitosan gel, buriti oil, and the associate gel is shown in Table 4.

### 3.4. Anti-Inflammatory Activity

The acute inflammatory process that occurs especially in the first three days after tissue damage is an innate immune response of the organism, in which the neutrophils produce free radicals to kill microorganisms and eliminate cellular debris by phagocytosis [17,37]. The inflammatory reaction is important because it delimits the injured region and prevents the spread of infections and pathogenic microorganisms. However, the intensity of the inflammatory response to eliminate pathogenic species and dead cells may be able to damage adjacent normal tissues. The clinical signs observed during the acute inflammatory process are redness, heat, pain, and swelling [32]

The literature describes that chitosan could inhibit or reduce the inflammatory cell infiltrate and that buriti oil has anti-inflammatory properties. The action of buriti oil is related to its antioxidant activity, which eliminates hydroxyl radicals derived from activated leukocytes in the site of injury. It also promotes inhibition of nitric oxide (NO) and, therefore, reduces the inflammation process [17,38]. It is known that the use of anti-inflammatory drugs available on the market can promote various side effects, therefore, the chitosan gel associated with buriti oil constitutes an interesting alternative material [32].

Chitosan gels, buriti oil, and chitosan gel with buriti oil showed inhibitory enzymatic effects. The effect was tested with the enzyme hyaluronidase, which exists naturally in the dermis and acts by depolymerizing hyaluronic acid, a component of the extracellular matrix, that presents chemical structure similar to that of chitosan. The tested materials showed anti-inflammatory activity, with enzyme inhibition percentages higher than 15% for all materials. There were no significant differences between them, but the percentage of inhibition for CGB was found to be intermediate in relation to the two starting materials (Table 5).

### 3.5. Healing Activity

#### 3.5.1. Macroscopic Analysis of Injuries

The healing of injured skin is regulated by several physiological and biochemical parameters, which act together to promote tissue repair. This process occurs naturally in the body, but the stimulation of appropriate drugs can happen faster and more efficiently [39,40,41].

Selecting a material that has the prerequisites of good healing is not easy. Most of the materials do not present all the desired properties. Chitosan gel activates macrophages, reduces inflammation time, and stimulates fibroblast proliferation and collagen deposition. However, pure chitosan gel does not have the same effect as commercially available ointments [42,43].

**Figure 4:**
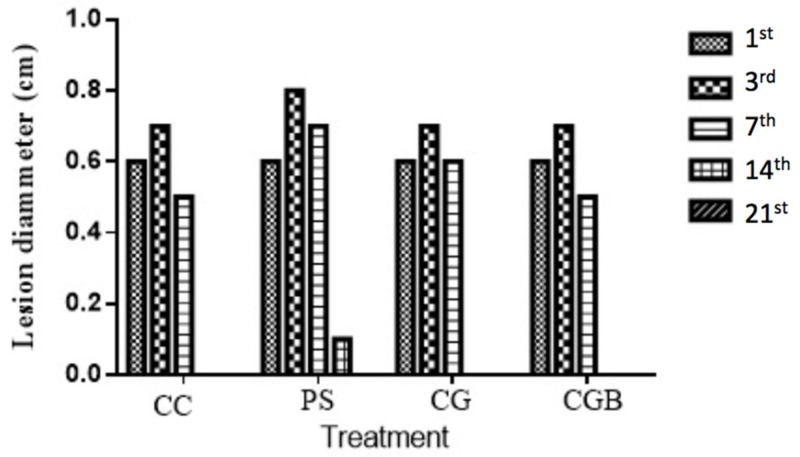
Evolution of the diameter of the skin lesion in mice on days on 3rd, 7th, 14th, and 21st days of treatment with Physiological Saline (PS), Collagenase *Clostridium histolyticum* ointment (CC), pure chitosan gel (CG), and chitosan gel with buriti oil (CGB).

Considering this limitation, the association of chitosan gel with biologically active molecules is an alternative to overcome this limitation and improve the healing action of this material by a synergic effect. In this work, the chitosan gel was associated with buriti oil, since the oil possesses antioxidant action. The evolution of the wound retraction process can be observed in Figure 4.

Macroscopic analysis of mice skin lesions, treatment using saline (negative control), collagenase (ABBOTT, Chicago, IL, USA) (positive control), pure gels, and oil-associated gel revealed the evolution of wound tissue repair with hemorrhage in the treated groups with SF, PC, and CG on the third and seventh day of treatment. This is detrimental to healing because it can compromise cell proliferation and promote bacterial growth [44,45].

All groups showed a larger lesion size on the third day due to the inflammatory process characteristic of this phase that delimits the injured region. The groups treated with CGB and collagenase^®^ ointment presented higher retraction rates from the seventh day.

Only in the group treated with SF did not heal the wound on the 14th day, and it had the lesion size still 0.1 cm. It can be explained by the presence of moderate inflammatory foci, while in the other groups healing was more effective.

In the first week of treatment, the presence of exudate was observed, especially in the SF, CG, and PC groups. All groups had the wound fully closed on the 21st day, indicating complete healing.

Chitosan gel is an active healing material that aids the complex process of reepithelization. Some studies prove that this material accelerates healing by stimulating the intense proliferation of fibroblasts and angiogenesis, besides preventing the development of infections. The association of this material with buriti oil may have accelerated the healing action, probably due to the high antioxidant activity and chemical composition of the oil [46].

#### 3.5.2. Qualitative Histopathological Evaluation

Histopathological evaluation was performed on days 1st, 3rd, 7th, 14th, and 21st of treatment. The histological structure of the skin on the first day showed the presence of multilayered keratinizing squamous epithelium, with the unmodified connective tissue of the dermis constituted by the *stratum papillare* and the *stratum reticulare*. These morphological characteristics indicate that there was no pre-existing injury to the injured region before the beginning of the procedure of wound excision/treatment.

**Figure 5:**
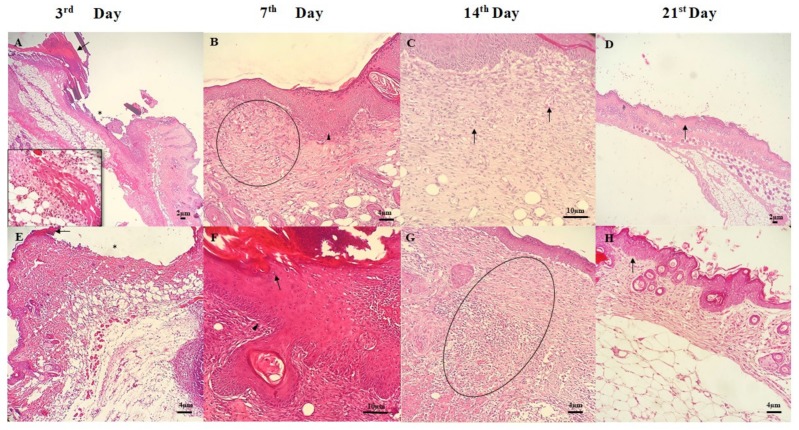
Photomicrographs of skin wounds on 3rd, 7th, 14th, and 21st day of treatment for groups treated with chitosan gel (**A**–**D**) and chitosan gel associated with buriti oil (**E**–**H**). Hematoxylin-Eosin (H.E.) stain (Scale bar: 2µm, 4 µm, and 10 µm). **A** and **E**: Skin ulcer with epidermis showing a large gap between the edges of the lesion (*), fibrin clot (arrows), and inflammatory infiltrate of polymorphonuclear cells in the dermis (detail). **B** and **F**: Continuing reepithelialization of the epidermis showing thickening (acanthosis—arrowheads), with keratinocytes meet in the midline beneath the surface scab in **F** (arrow) and presence of neoformed vessels in granulation tissue in the dermis (circle). **C** and **G**: There are epidermal reepithelialization and extensive granulation tissue formation, with neoformed vessels (arrows), and an inflammatory process located in the deepest dermis (circle). **D** and **H**: continuing reepithelialization of the epidermis and wound contraction (arrows).

On the third day of treatment, the animals treated with chitosan gel still showed an inflammatory infiltrate of polymorphonuclear cells (neutrophils) at the incision margin, migrating toward the fibrin clot (Figure 5A). In fact, the clot aid to stop bleeding and acts as a scaffold for the proliferation/migrating of epithelial cells, which are attracted by growth factors, cytokines, and chemokines released into the area. On the other hand, neutrophils release proteolytic enzymes to remove cellular debris, fibrin or other foreign material, and aid the epithelial cells from both edges to migrate and proliferate alongside the dermis, depositing the basement membrane components. The animals treated with chitosan gel associated with buriti oil showed similar characteristics, with the presence of fibrin and a smaller amount of neutrophils concentrated in the ulcer region and more diffuse in the dermis. However, the presence of macrophages infiltrate with mild intensity in the dermis was observed, besides a few eosinophils and neoformed capillaries, indicating that granulation tissue progressively invades the incision space (Figure 5A,E).

The seventh day of treatment was characterized by epithelial reepithelialization in the CG group. In the wound area, it was possible to identify the thickening of the epidermis (acanthosis) caused by the proliferation of keratinocytes. Additionally, the deposition of collagen fibers was more abundant with the presence of neovascularization, fibroblast proliferation, and extracellular matrix (ECM) deposition. Macrophages were the main type of Inflammatory cells in the dermis observed. The main difference for the group treated with CGB was the incomplete reepithelization, presence of crust, many neoformed vessels, and degenerate neutrophils (Figure 5B,F).

In the CG group, there was a decrease in inflammation on the 14th day, with gradually increasing collagen deposition within the incisional scar and the regression of vascular channels. Some macrophages and fibroblasts were observed. The granulation tissue had a little more mature appearance, filling the entire area of the lesion. There was no cellular debris, and it was still possible to observe acanthosis and neoformed vessels (Figure 5C,G).

The lesion in the animals of the CGB group on the 14th day of treatment presented complete reepithelization in the epidermis. In the upper dermis, collagen fibers were observed in horizontal arrangement with some orientation and also hair follicles. Moreover, there is extensive granulation tissue formation with the infiltration of mononuclear cells located in the deepest dermis (Figure 5G). It is common knowledge that inflammation is more intense in large tissue defects because a greater volume of necrotic debris, exudate, and fibrin must be removed. Thus, large defects have a greater potential for secondary infection, delaying wound healing.

All animals were fully healed on the 21st day, besides total reepithelialization, completely recovered dermis, and few newly formed vessels (Figure 5D). In the case of animals treated with CGB, the horizontal organization of collagen fibers proves the greatest contraction of the wound. For this group, it was also found that the fibroblasts had returned to spindle-shaped cell morphology, and the mature granulation tissue was composed of dense collagen fibers. The epidermis has regained its normal thickness and architecture (Figure 5H).

## 4. Conclusions

Analysis of volatile compounds and fatty acids from buriti oil showed that the chemical composition of the material is responsible, especially for its antioxidant action. The developed gel was easy to obtain and presented proper thermal stability. An important consequence of this association was the synergism observed against bacteria, particularly against gram-negative strains. Besides the antimicrobial activity, the chitosan gel with buriti showed antioxidant, anti-inflammatory, healing activity, and an adequate wound retraction rate, indicating that the CGB is efficient to be used treating skin lesions. However, texture assays and release studies are required for commercial production.

## Figures and Tables

**Table 1:** Minority volatile compounds in buriti oil.

Retention Time (min)	Volatile Compounds Identified (mg/L)	[ ] mg/L ± SD
13.543	4-nonanol	239.880 ± 0.000
28.288	2-phenylethanol	4839.517 ± 2371.759
29.492	b-ionone	2362.144 ± 603.889
39.699	ethyl hexadecanoate	50,653.380 ± 5430.406
45.788	(Z)-9-octadecenoic acid ethyl ester	137,693.500 ± 15,294.920
45.994	2-hydroxy-1-(hydroxymethyl)-(Z)-9-octadecenoic acid ethyl ester	5745.419 ± 505.733
51.527	Ecosyl ester oleic acid	1415.337 ± 160.063
56.231	Hexadecanoic acid (palmitic)	852,395.300 ± 32,492.960
61.736	(Z)-9-octadecenoic acid	3,803,073.000 ± 201,189.900

**Table 2:** Fatty acids composition of buriti oil.

Carbon	Compound	Percentage (%)
**-**	**Saturated Fatty Acids**	**-**
C8:0	Caprylic Acid	0.0045
C12:0	Lauric acid	0.012
C14:0	Mystic acid	0.071
C15:0	Pentadecylic Acid	0.0675
C17:0	Margaric Acid	0.086
C16:0	Palmitic acid	20.134
C18:0	Stearic acid	0.9465
C20:0	Arachidic acid	0.085
**-**	**Monounsaturated**	**-**
C16:1	Palmitic acid	0.3635
C17:1	Margaric acid	0.0935
C18:1n9c+t	Elaidic acid and oleic acid	75.43
C20:1	Gondoic acid	0.5745
**-**	**Polyunsaturated**	**-**
C18:2n6c	Linoleic acid	1.1955
C18:3n3	Linolenic acid	0.891

**Table 3:** Minimum Inhibitory Concentration (MIC) (mg/mL) for pure chitosan gels (CG) and associated with buriti oil (CGB) and buriti oil (OB) against *S. aureus* and *K. pneumoniae.*

Materials	MIC (mg/mL)	
	*S. aureus* ATCC 43300	*K. pneumoniae* ATCC 13883
CG	10.00 ± 0.00	10.00 ± 0.00
OB	nd*	nd*
CGB	5.00 ± 0.00	3.75 ± 1.76

*nd = not determined.

**Table 4:** Antioxidant activity assessed by the β-carotene method for pure chitosan gel (CG) buriti oil (OB) and gel with buriti oil (CGB) gels.

Material	[ ] mg/mL	β-carotene
		Average ± SD (µmol/LBHA)
CG	10.0	0.232 ± 0.096
OB	2.0	1.131 ± 0.355
CGB	10.0	1.703 ± 0.236

**Table 5:** Inhibition of hyaluronidase activity by gels samples of pure chitosan (CG) and associated with buriti oil (CGB) and buriti oil (OB).

Sample	[ ] mg/mL of Sample	Percent Inhibition (%)
CG	30.0	15.53 ± 0.65
OB	2.5	16.86 ± 1.00
CGB	30.0	16.63 ± 0.66
